# Effect of soil aeration on root morphology and photosynthetic characteristics of potted tomato plants (*Solanum lycopersicum*) at different NaCl salinity levels

**DOI:** 10.1186/s12870-019-1927-3

**Published:** 2019-07-29

**Authors:** Yuan Li, Wenquan Niu, Xiaoshu Cao, Jingwei Wang, Mingzhi Zhang, Xiaohui Duan, Zhenxing Zhang

**Affiliations:** 1 Northwest Land and Resources Research Center, Shaanxi Normal University, Xi’an, 710119 Shaanxi China; 2 Institute of Soil and Water Conservation, Northwest A&F University, No.26 Xinong Road, Yangling, Shaanxi Province 712100 People’s Republic of China; 3 Institute of Water-saving Agriculture in Arid Areas of China (IWSA), Northwest A&F University, Yangling, 712100 Shaanxi China; 4 Institute of Soil and Water Conservation, Chinese Academy of Sciences and Ministry of Water Resources, Yangling, 712100 Shaanxi China; 5 Key Laboratory of Vegetation Ecology, Ministry of Education, Institute of Grassland Science, Northeast Normal University, Changchun, 130024 Jilin Province China; 6 State Environmental Protection Key Laboratory of Wetland Ecology and Vegetation Restoration, School of Environment, Northeast Normal University, Changchun, 130117 Jilin Province China

**Keywords:** Tomato, Soil aeration, NaCl salinity, Root morphology, Photosynthetic characteristics

## Abstract

**Background:**

Salt stress is one of the environmental factors that greatly limits crop production worldwide because high salt concentrations in the soil affect morphological responses and physiological and metabolic processes, including root morphology and photosynthetic characteristics. Soil aeration has been reported to accelerate the growth of plants and increase crop yield. The objective of this study was to examine the effects of 3 NaCl salinity levels (28, 74 and 120 mM) and 3 aeration volume levels (2.3, 4.6 and 7.0 L/pot) versus non-aeration and salinity treatments on the root morphology, photosynthetic characteristics and chlorophyll content of potted tomato plants.

**Results:**

The results showed that both aeration volume and salinity level affected the root parameters, photosynthetic characteristics and chlorophyll content of potted tomato plants. The total length, surface area and volume of roots increased with the increase in aeration volume under each NaCl stress level. The effect was more marked in the fine roots (especially in ≤1 mm diameter roots). Under each NaCl stress level, the photosynthetic rate and chlorophyll content of tomato significantly increased in response to the aeration treatments. The net photosynthetic rate and chlorophyll a and t content increased by 39.6, 26.9, and 17.9%, respectively, at 7.0 L/pot aeration volume compared with no aeration in the 28 mM NaCl treatment. We also found that aeration could reduce the death rate of potted tomato plants under high salinity stress conditions (120 mM NaCl).

**Conclusions:**

The results suggest that the negative effect of NaCl stress can be offset by soil aeration. Soil aeration can promote root growth and increase the photosynthetic rate and chlorophyll content, thus promoting plant growth and reducing the plant death rate under NaCl stress conditions.

## Background

Environmental stresses such as those caused by acid/alkaline/saline soils are the main restricting factors on crop production worldwide [1]. Salinity is one of the major abiotic stress factors that adversely affects crop plant yield and mass. Tuteja’s research [2] found that approximately 23% of cultivated land was affected by salinity. Ashraf’s research [3] indicates that the potential yield losses from salinity are estimated at 20%. The impact of environmental stress around the world caused by salinity is constantly expanding in many parts of the world, particularly in arid and semiarid areas, because agriculture expanding to these regions with modern irrigation practices will exacerbate secondary salinization [4–6]. These problems also occur in China, where it was estimated that approximately 3,693 hm^2^ of land is saline [7]. It is well known that salinity may have deleterious effects on plant growth, and crop production is low in saline soils. Low concentrations of salt can induce growth retardation in non-halophytic plants, while high concentrations can even result in the death of the plant [8–10]. This is mainly due to salt toxicity leading to a decrease in plant water holding capacity, imbalances in nutrient uptake, and the toxicity of ions to plant roots and photosynthesis [11, 12].

Currently, the salinity in soils is mainly caused by three kinds of cations (Na^+^, Ca^2+^, Mg^2+^) and four kinds of anions (CO_3_^2−^, HCO_3_^−^, Cl^−^, SO_4_^2−^) [13]. NaCl is the most universally distributed type of salt at present and does substantial harm. NaCl has been shown to inhibit uptake and transport of N, P, K and Ca [14]. Additionally, NaCl stress leads to reductions in Zn uptake by plant roots due to Na^+^ competition at the root surface [15]. More remarkably, most saline land suffers from waterlogging [16] or soil compaction [17]. Both of these conditions easily cause hypoxia stress (low O_2_).

Plant roots require sufficient oxygen for the production of ATP from sugars. Through aerobic respiration, 1 mol glucose can produce 38 mol ATP. However, under anaerobic conditions, only 2 mol of ATP can be produced. Therefore, plant roots cannot obtain enough energy to maintain health and normal physiological activity under hypoxic conditions. Second, the metabolites (such as ethanol, lactate, H_2_O_2_, etc.) accumulated during hypoxia are hazardous materials for plants themselves [18, 19]. In addition, the negative impacts of salinity are severe when the rhizosphere is exposed to hypoxia. The root uptake of Na^+^ and Cl^−^ increases with decreasing O_2_ concentration in the rhizosphere [20]. NaCl stress causes greater increases in Na^+^ and Cl^−^ concentrations in the shoots and greater decreases the concentrations of K^+^ [21].

Tomato is a principal vegetable crop due to its worldwide consumption and is adapted to a variety of climates, from temperate to tropical and from arid to humid areas [22]. However, salt stress is a main factor that limits the fruit yields of tomato, because salinity seriously disrupts tomato plant growth at all stages of growth, thus reducing fruit production.

Photosynthesis is the first process to be compromised by salinity. Salt stress leads to a reduction in leaf water potential and both stomatal resistance and mesophyll resistance to gas flows rises, with a subsequent limitation of photosynthetic activity [23, 24]. The reduction of stomatal conductance and photosynthesis under salt stress reduces leaf growth and dry matter [25]. The root system is the main part of the plant to experience soil salinity and likely plays a vital role in coping with soil salts. How salts and hypoxia affect root growth and morphology is of great importance to elucidate the mechanism of plant adaptation processes. Previous studies have shown that salinity reduced the root length density of tomato and the root dry matter reduction under salinity was combined with a root/shoot ratio increase [26, 27].

Artificial aeration has been reported to increase dissolved O_2_ content and ameliorate the negative effects of hypoxia stress [28–34]. Previous studies strongly suggest that increased access to O_2_ by aeration has positive impacts on plant growth and physiology. In sandy soil, harvest data showed that bell pepper plants irrigated with aerated water produced 39% greater weight, than plants irrigated with non-aerated water [35]. Since aeration increases the concentration of O_2_ in the root zone, it is reasonable to infer that the observed positive effects are indirectly linked to the response of the plant root and photosynthetic system.

In Northwest China, hypoxic conditions are usually accompanied by salt stress. With a constantly increasing population, the shortage of land resources has become an urgent problem in China [36]. In recent years, in order to produce more fruits, farmers have been forced to plant tomatoes in saline land [37]. Nevertheless, most of the previous studies have dealt with artificial aeration and salinity separately, and little is known about the potential interaction between these two factors in tomato plants. The objective of this study was to quantify the effects of non-aeration versus 3 aeration volumes in potted tomato plants at 3 NaCl stress levels. Specifically, we examined data on plant growth, leaf photosynthetic rate and root morphology.

## Results

### Growth rate

The effects of different aeration treatments on the growth rates of plant height and stem diameter under certain NaCl conditions are shown in Table 1. The results showed that root zone aeration had a significant effect on the growth rates of plant height from 0 to 30 DAT and of stem diameter from 70 to 90 DAT. NaCl stress had a significant effect on the growth rates of plant height from 0 to 70 DAT and of stem diameter from 0 to 30 and 70–90 DAT. The ANOVA F-value showed that the interaction of NaCl stress and aeration level was not significant for plant growth rate at 0–90 DAT. Throughout the growth period, the plant height growth rate exhibited a trend of initial increase (0–70 DAT) followed by a decrease (70–90 DAT) under CK and S_1_ treatments, and the plant height growth rate increased with tomato growth (0–90 DAT) under S_2_ treatment. Except for the stem diameter growth rate at 50–70 DAT, all the treatment combinations of aeration volume and NaCl stress were significant at the 5% level. From 0 to 70 DAT, the plant height growth rate decreased with increasing NaCl stress under each aeration treatment. The highest plant height growth rate was observed in the CK, S_1_A_2_ and S_2_A_2_ treatments during 0–50, 50–70, and 70–90 DAT, respectively, and the highest stem diameter growth rate was observed in the CK, S_1_A_3_ and S_3_A_2_ treatments during 0–30, 30–50, and 70–90 DAT, respectively.

**Table 1:** plant growth rate of potted single tomato plants measured at 0, 30, 50, 70 and 90 days after transplanting (DAT) for the 4 aeration levels at the 3 NaCl stress levels

Treatments	Growth rate of plant height/ (cm/Day)				Growth rate of stem diameter/ (mm/Day)			
	0-30DAT	30-50DAT	50-70DAT	70-90DAT	0-30DAT	30-50DAT	50-70DAT	70-90DAT
CK	0.99a	1.26a	1.80ab	0.84ab	0.79a	0.48ab	0.15a	0.54bcde
S_1_A_0_	0.74bcd	0.83abc	1.63abc	1.23ab	0.33bcd	0.24abc	0.49a	0.81abc
S_1_A_1_	0.83ab	0.76bcd	1.80ab	1.53ab	0.38bc	0.33abc	0.48a	1.06ab
S_1_A_2_	0.96a	1.01ab	1.85a	1.21ab	0.73a	0.32abc	0.47a	0.78abc
S_1_A_3_	0.78bc	1.06ab	1.34abcd	1.31ab	0.54ab	0.58a	0.41a	0.83abc
S_2_A_0_	0.48e	0.36de	1.19bcd	1.30ab	0.25 cd	0.08bc	0.67a	0.13e
S_2_A_1_	0.63cde	0.44cde	0.93def	1.63ab	0.35bcd	0.04c	0.61a	0.31cde
S_2_A_2_	0.62cde	0.48cde	1.39abcd	1.70a	0.40bc	0.41abc	0.35a	0.69abcd
S_2_A_3_	0.60de	0.49cde	1.05cde	1.15ab	0.32bcd	0.45abc	0.19a	0.32cde
S_3_A_0_	0.51e	0.24e	0.30 g	0.60b	0.09d	0.32abc	0.17a	0.30cde
S_3_A_1_	0.53e	0.29e	0.43 fg	0.87ab	0.23 cd	0.20abc	0.49a	0.19de
S_3_A_2_	0.57e	0.34de	0.48efg	1.20ab	0.26 cd	0.47abc	0.16a	1.11a
S_3_A_3_	0.55e	0.21e	0.29 g	0.94ab	0.23 cd	0.23abc	0.31a	0.44cde
F-value								
Aeration volume (A)	3.396*	0.637 ns	1.528 ns	0.755 ns	4.040*	2.685 ns	1.243 ns	3.446*
Salinity level (S)	37.611**	22.114**	31.761**	2.078 ns	19.615**	1.725 ns	1.970 ns	7.479**
A × S	0.827 ns	0.341 ns	0.521 ns	0.371 ns	0.861 ns	1.177 ns	0.866 ns	2.197 ns

Note: Values followed by different small letters in the same column meant significant differences at *p* < 5%. ANOVA F-value for main and interaction effects were not significant (ns) or significant at ≤5% (*) and ≤ 1% level (**)

### Total root length, surface area, volume and number of forks

The results of the ANOVA indicated that both aeration and salinity had significant effects on total root length, surface area and root volume of potted tomato plants (Table 2). Two-way interaction effects were highly significant on total root length, root surface area and number of forks, but not on the total root volume of the potted tomato plants. As shown in Table 2, with no aeration, plants under each NaCl stress level had significantly lower total root length, surface area, volume and number of forks than those in the non-NaCl stress treatment (CK). This indicates that the tomato root is sensitive to NaCl stress. Nevertheless, with no aeration, the root length, surface area, volume and number of forks of potted tomato plants under the S_1_ treatment were 17.8, 20.9, 22.0, and 37.2% higher than of those under the S_2_ treatment, respectively. The total root length, surface area, volume and number of forks increased with the increasing aeration volume under the S_1_ treatment. Although all the plants in the S_3_A_0_ treatment combination died, under the S_2_ and S_3_ treatments, root length, surface area, volume and number of forks of potted tomato plants showed first an increase and then a decrease when the aeration volume increased; under the A_2_ treatment, the 4 variables reached their maximum. Except in the S_2_A_1_ treatment combination, the root length, surface area, volume and number of forks under the S_1_ and S_2_ salinity levels with aeration treatment were higher than those under CK.

**Table 2:** total root length, surface area, volume and forks of potted single tomato plants measured at mature period (155 DAT) for the 4 aeration levels at the 3 NaCl stress levels

Treatments	Root length (cm/plant)	Root surface area (cm^2^/plant)	Root volume (cm^3^/plant)	forks
CK	8045de	717 cd	5.18bcd	63822de
S_1_A_0_	5644 fg	489ef	3.50def	37185 fg
S_1_A_1_	8505cde	851bc	7.06ab	82247 cd
S_1_A_2_	10272bc	916bc	6.63abc	100433bc
S_1_A_3_	15040a	1300a	9.16a	138341a
S_2_A_0_	6649ef	591de	4.27cde	51007ef
S_2_A_1_	7267ef	747 cd	6.34bc	71791de
S_2_A_2_	11516b	1034b	7.48ab	112764b
S_2_A_3_	9595bcd	850bc	6.23bc	83795 cd
S_3_A_0_	–	–	–	–
S_3_A_1_	1988	158	1.02	11289
S_3_A_2_	3923gh	333 fg	2.28ef	22047 g
S_3_A_3_	2697 h	198 g	1.21f	15745 g
F-value				
Aeration volume (A)	36.001**	22.764**	10.778**	29.807**
Salinity level (S)	67.687**	50.030**	25.847**	49.161**
A × S	9.798**	5.559**	2.118 ns	5.852**

Note: Values followed by different small letters in the same column meant significant differences at *p* < 5%. ANOVA F-value for main and interaction effects were not significant (ns) or significant at ≤5% (*) and ≤ 1% level (**).Under the S_3_A_0_ treatment plants all died, So the S_3_A_0_ row is empty. Multiple comparison can’t be performed for S_3_A_1_ treatment because only 1 plant survived

### Root length, surface area, and volume distribution by diameter

The main treatment and interaction effects on root length, surface area and volume by range of diameter are shown in Table 3. The aeration volume had no significant effect on the root surface area for diameters over 2 mm. The ANOVA showed that both salinity and soil aeration treatments significantly affected root length and surface area distribution by root diameter of potted tomato plants (Table 3). The experimental results showed that the aeration volume and the salinity level had significant effects on root length and surface area distribution for diameters 0 < D ≤ 0.1, 0.1 < D ≤ 0.2, 0.2 < D ≤ 0.5, 0.5 < D ≤ 1.0 and 1.0 < D ≤ 2.0 but had no significant effect on root volume for diameters over 2 mm. All two way interactions effects on root length, surface area and volume of tomato plants were highly significant for diameters 0 < D ≤ 0.1, 0.1 < D ≤ 0.2, 0.2 < D ≤ 0.5 mm, but not on root length, surface area and volume for diameters over 0.5 mm.

**Table 3:** Two-way ANOVA of Aeration volume (A), Salinity level (S) and the A × S interaction on root length (cm/plant), surface area (cm^2^/plant) and volume (cm^3^/plant) distribution by root diameter

	Root diameter (D) mm	Aeration volume (A)	Salinity level (S)	A × S
Root length	0–0.1	34.642**	50.032**	7.990**
	0.1–0.2	23.235**	47.477**	8.981**
	0.2–0.5	16.141**	43.765**	5.879**
	0.5–1.0	12.696**	28.400**	2.458 ns
	1.0–2.0	7.176**	12.649**	0.805 ns
	>2.0	3.752*	4.764*	0.301 ns
Root surface area	0–0.1	34.675**	51.435**	8.355**
	0.1–0.2	23.132**	48.499**	9.162**
	0.2–0.5	15.997**	43.234**	5.369**
	0.5–1.0	12.492**	27.429**	2.377 ns
	1.0–2.0	6.941**	12.180**	0.762 ns
	>2.0	2.894 ns	4.028*	0.320 ns
Root volume	0–0.1	35.306**	53.769**	8.872**
	0.1–0.2	22.956**	49.463**	9.337**
	0.2–0.5	15.728**	42.319**	4.867**
	0.5–1.0	12.253**	26.422**	2.289 ns
	1.0–2.0	6.704**	11.704**	0.724 ns
	>2.0	1.794 ns	3.025 ns	0.336 ns

Note: ANOVA F-value for main and interaction effects were not significant (ns) or significant at ≤5% (*) and ≤ 1% level (**)

The results of the ANOVA indicated that the treatment effects of aeration volume and salinity level and their interaction all had a significant influence on these morphological observations. Treatment effects on these morphological traits were more marked at the lower diameter ranges, and the influence of salinity is larger than that of aeration volume (Table 3).

**Fig. 1:**
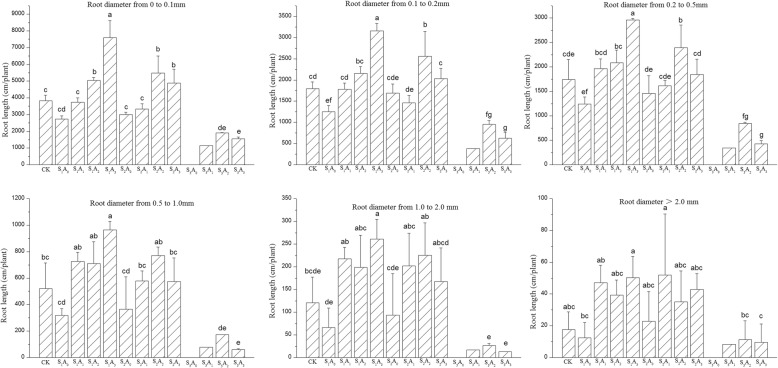
Effects of NaCl stress and soil aeration on root length (cm/plant) distribution by root diameter for potted tomato. Columns with the same letter represent values that are not significantly different at the 5% level of probability according to the Duncan’s new multiple-range test. Under the S3A0 treatment plants all died, So the S3A0 column is empty. Multiple comparison can’t be performed for S3A1 treatment because only 1 plant survived

The response pattern for root length by diameter was identical to that for root length. Under the no-aeration treatment, NaCl stress resulted in decreased root length at each diameter range. Nevertheless, the root lengths were not significantly different from the S_1_ and S_2_ marginal means (Fig. 1). Under the S_1_ treatment, root length increased with increasing aeration volume for root diameters 0 < D ≤ 0.1 mm, 0.1 < D ≤ 0.2 mm, 0.2 < D ≤ 0.5 mm and 0.5 < D ≤ 1.0 mm, The values with no aeration were 2736, 1251, 1239 and 319 cm, and with V_3_ were 7598, 3155, 2958 and 964 cm, which were 178, 152, 139 and 202% greater than the values for the no-aeration treatment for root length distribution at each diameter range, respectively. Under the S_2_ treatment, the root length for diameters 0 < D ≤ 0.1 mm, 0.1 < D ≤ 0.2 mm, 0.2 < D ≤ 0.5 mm, 0.5 < D ≤ 1.0 mm and 1.0 < D ≤ 2.0 mm initially increased and then decreased as the soil aeration volume increased and reached a maximum under the A_2_ treatment. The root length values for potted tomato plant aeration under A_2_ treatment for each diameter were 5476, 2558, 2392, 771 and 226 cm, respectively. Under heavy NaCl stress, the no-aeration treatment plants all died. Nevertheless, the root length distribution at each diameter range initially increased and then decreased as the aeration frequency increased, reaching a maximum under the A_2_ treatment. The maximum values for each diameter range were1900, 950, 845, 173, 27 and 11 cm, respectively.

**Fig. 2:**
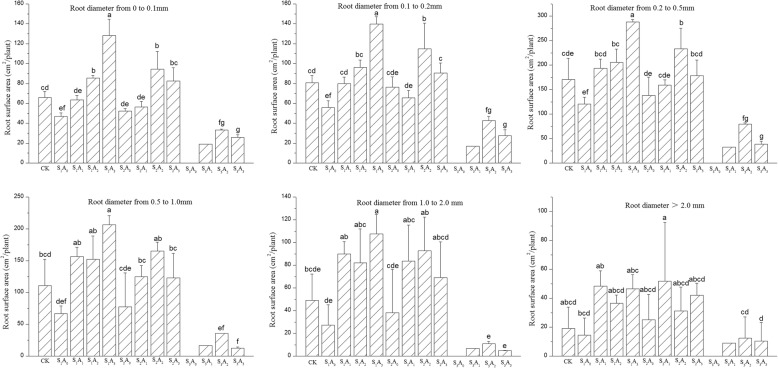
Effects of NaCl stress and soil aeration on root surface area (cm2/plant) distribution by root diameter for potted tomato. Columns with the same letter represent values that are not significantly different at the 5% level of probability according to the Duncan’s new multiple-range test. Under the S3A0 treatment plants all died, So the S3A0 column is empty. Multiple comparison can’t be performed for S3A1 treatment because only 1 plant survived

**Fig. 3:**
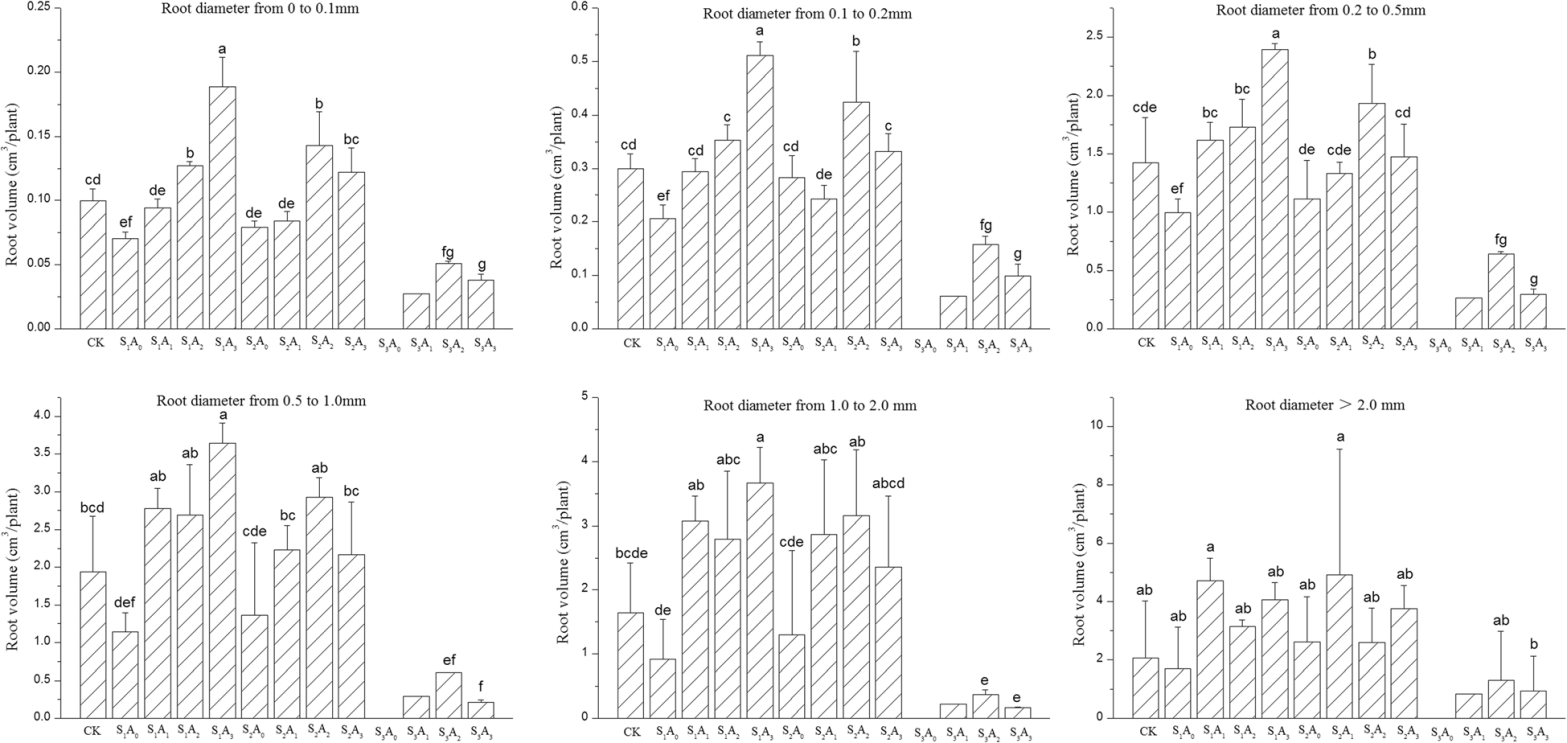
Effects of NaCl stress and soil aeration on root volume (cm3/plant) distribution by root diameter for potted tomato. Columns with the same letter represent values that are not significantly different at the 5% level of probability according to the Duncan’s new multiple-range test. Under the S3A0 treatment plants all died, So the S3A0 column is empty. Multiple comparison can’t be performed for S3A1 treatment because only 1 plant survived

Figures 2 and 3 show a similar response pattern for root surface area and volume for each diameter (Table 3). In general, soil aeration improved the root surface area and volume at each diameter range. Nevertheless, salinity stress decreased the root surface area and volume for each diameter, especially in the S_3_ treatment. The highest values of root surface area for root diameters of 0 < D ≤ 0.1 mm, 0.1 < D ≤ 0.2 mm, 0.2 < D ≤ 0.5 mm and 0.5 < D ≤ 1.0 mm were observed in the S_1_A_3_ treatment combination, and the values for each diameter were 128, 140, 288 and 206 cm^2^, respectively (Fig. 2). Similarly, the root surface area initially increased, and then decreased when the soil aeration volume increased and reached a maximum under the A_2_ treatment for diameters 0 < D ≤ 0.1 mm, 0.1 < D ≤ 0.2 mm, 0.2 < D ≤ 0.5 mm, 0.5 < D ≤ 1.0 mm and 1.0 < D ≤ 2.0 mm. Similarly, increasing aeration volume progressively increased root volume for root diameters 0 < D ≤ 0.1 mm, 0.1 < D ≤ 0.2 mm, 0.2 < D ≤ 0.5 mm and 0.5 < D ≤ 1.0 mm under S_1_ treatment (Fig. 3). Differences between the marginal means were significant at the 5% level, and the values for no aeration were 0.07, 0.21, 1.00 and 1.15 cm^3^ for each diameter range, respectively. Values for each diameter range V_3_ treatment were 0.19, 0.51, 2.39, and 3.65, which were 167, 148, 140, and 218% greater, respectively, than the value for the no-aeration treatment.

### Photosynthetic characteristics

**Fig. 4:**
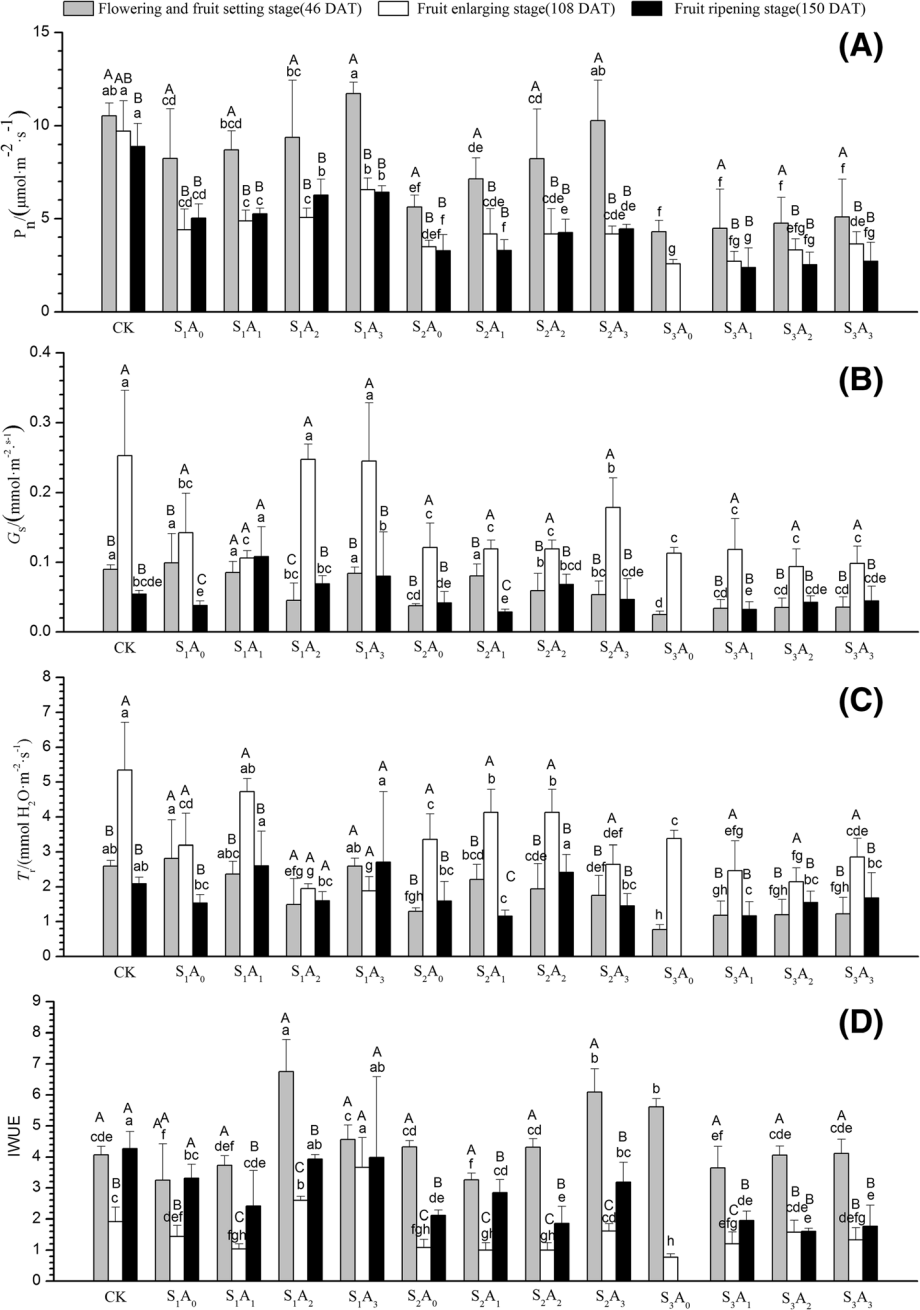
Effects of NaCl stress and soil aeration on (a) net photosynthetic rate (Pn), (b) stomatal conductance (Gs), (c) transpiration rate (Tr) and (d) instantaneous water use efficiency at different growth stages for potted tomato. Columns with the same letter represent values that are not significantly different at the 5% level of probability according to the Duncan’s new multiple-range test for different treatments at same growth stage, and same capital letter represent values that are not significantly different at the 5% level of probability according to the Duncan’s new multiple-range test at different growth stage for the same treatments. At the fruit ripening stage, under the S3A0 treatment plants all died, so the column is empty

The results of marginal means for *P*_n_ are shown in Fig. 4a, ranging from 2.4 to 11.7 μmol CO_2_ m^− 2^ s^− 1^. *P*_n_ increased with increasing aeration volume at for 46, 108 and 150 DAT at each salinity level. Compared with other treatments, *P*_n_ reached a maximum under the S_1_A_3_ treatment at 46 DAT, whereas at 108 and 150 DAT, *P*_n_ reached a maximum under CK treatment. Measurements of *P*_n_ under the S_1_A_3_ treatment were 42, 49 and 28% higher than those under the S_1_A_0_ treatment at 46, 108, and 150 DAT, respectively. Under the S_2_A_3_ treatment, measurements of *P*_n_ 83, 20 and 36% higher than those under the S_2_A_0_ treatment at 46, 108, and 150 DAT, respectively. Nevertheless, *P*_n_ decreased as salinity increased at each aeration level. The *G*_s_ under the S_1_ salinity level with aeration treatment was higher than that with no aeration at 108 and 150 DAT (Fig. 4b). Except for the S_1_A_3_ treatment combination, potted tomato plants under all the treatments at 108 DAT showed the highest *T*_r_ values (Fig. 4c). At 46 DAT, the *T*_r_ of potted tomato plants decreased with increasing salinity level. The *IWUE* of potted tomato plants was lower at 108 DAT than at 46 and 150 DAT (Fig. 4d). S_1_A_2_ treatment at 46 DAT, S_1_A_3_ treatment at 108 DAT and CK treatment at 150 DAT showed the highest values for *IWUE*. The *IWUE* under S_1_A_2_ and S_1_A_3_ treatments was higher than that of CK at each measurement.

### Chlorophyll content

Table 4 shows that all the combinations had significant impacts on total chlorophyll (Chl. t), chlorophyll a (Chl. a) and chlorophyll b (Chl. b) contents at 46 DAT (fruit setting stage) and 108 DAT (fruit enlargement stage). The Chl. a content was higher at 108 DAT than at 46 DAT under CK and each aeration treatment with S_1_ and S_2_. The results showed that, except in for the S_3_A_0_ and S_3_A_1_ treatment combinations, the contents of Chl. a, Chl. b and Chl. t at 108 DAT were higher than those at 46 DAT. As shown in Table 4, at both 46 and 108 DAT, the content of Chl. a increased with increasing aeration volume at each salinity level. Nevertheless, all the values under each salinity treatment were lower than that of CK. Similarly, the contents of Chl. b at 46 DAT increased with increasing aeration volume at each salinity level, with CK having the maximum value of 0.81 mg·g^− 1^ and S_3_A_0_ having the minimum value of 0.38 mg·g^− 1^. At 108 DAT, maximum values of both Chl. b and Chl. t contents were observed in the S_2_A_3_ (Chl. b = 1.77, Chl. t = 4.22 mg·g^− 1^) and S_3_A_3_ (Chl. b = 1.22, Chl. t = 3.18 mg·g^− 1^) treatment combinations. Contents of Chl. t increased when the soil aeration volume increased, and reached a maximum under the S_1_A_2_, S_1_A_3_ and S_2_A_3_ treatment combinations at 46 DAT (Table 4). The ANOVA results showed that while salinity was significant for Chl. a, Chl. b and Chl. t at both 46 and 108 DAT, the aeration volume was not significant. The effect of aeration volume on promoting Chl. a, Chl. b and Chl. t at 46 DAT is more significant than that at 108 DAT. Interaction analysis found that combinations of aeration volume and salinity level had no significant impacts on the contents of Chl. a, Chl. b and Chl. t at both 46 and 108 DAT.

**Table 4:** chlorophyll a, chlorophyll b and total chlorophyll of potted single tomato plants measured at 46 and 108 days after transplanted (DAT) for the 4 aeration levels at the 3 NaCl stress levels

Treatments	Chl. a / (mg·g^−1^)		Chl. b / (mg·g^−1^)		Chl t/ (mg·g^−1^)	
	46DAT	108DAT	46DAT	108DAT	46DAT	108DAT
CK	2.27a	2.94a	0.81a	1.14abc	3.08a	4.07a
S_1_A_0_	1.63abcd	2.12abc	0.55bcde	1.22abc	2.18abc	3.35ab
S_1_A_1_	1.81abcd	2.29abc	0.67abcd	1.21abc	2.48ab	3.51ab
S_1_A_2_	2.09a	2.41ab	0.70abc	1.27abc	2.78a	3.68ab
S_1_A_3_	2.16a	2.60ab	0.78ab	0.99abc	2.93a	3.59ab
S_2_A_0_	1.75abcd	1.83abc	0.61abcde	1.13abc	2.36abc	2.96abc
S_2_A_1_	1.92ab	2.27abc	0.65abcd	1.53ab	2.57ab	3.81ab
S_2_A_2_	1.88abc	2.36ab	0.66abcd	1.00abc	2.54ab	3.35ab
S_2_A_3_	2.04a	2.45ab	0.69abc	1.77a	2.73a	4.22a
S_3_A_0_	1.14d	0.61c	0.38e	0.47c	1.52c	1.08c
S_3_A_1_	1.21 cd	1.03bc	0.51cde	0.60c	1.72bc	1.63bc
S_3_A_2_	1.27bcd	1.26abc	0.44de	0.83bc	1.71bc	2.09abc
S_3_A_3_	1.65abcd	1.97ab	0.58abcde	1.22a	2.23abc	3.18a
F-value						
Aeration volume (A)	2.480 ns	1.481 ns	2.880 ns	1.716 ns	2.637 ns	2.257 ns
Salinity level (S)	10.369**	6.299**	10.403**	4.351*	10.785**	7.798**
A × S	0.255 ns	0.180 ns	0.280 ns	2.111 ns	0.236 ns	0.579 ns

Note: Values followed by different small letters in the same column meant significant differences at p < 5%. ANOVA F-value for main and interaction effects were not significant (ns) or significant at ≤5% (*) and ≤ 1% level (**)

## Discussion

There are several factors that affect plant growth, such as soil moisture, nutrients, salinity, temperature, oxygen content and the mechanical impedance of the plant rhizosphere [38]. Our investigation has shown that NaCl salinity inhibited tomato growth. The *P*_n_ and root morphology of tomato plants significantly increased in response to aeration under NaCl salinity conditions. Therefore, soil aeration strongly promotes plant growth.

### NaCl salinity inhibited tomato growth

In response to NaCl toxicity, roots can change their architecture [1, 12, 39, 40]. The results of this experiment showed that root morphology (length, surface area, volume, number of forks and tips) decreased significantly with increasing concentrations of NaCl (28–120 mM) when compared with the root morphology under the no-NaCl treatment, especially in thin (D ≤ 0.5 mm) roots. Our result is in accordance with previous studies of root morphology response to NaCl salt stress in tomato, rice, euonymus and wheat [40–43]. Various reasons for the reduced root growth under salt stress are possible. High concentrations of salts in soils account for large decreases in the length and surface area of plant roots and are accompanied by a low uptake of salinity, water, nutrients, etc. Lin [44] reported that NaCl salt stress increased cell-wall peroxidase activity and H_2_O_2_ levels. Exogenous H_2_O_2_ and peroxidase were found to inhibit plant root growth.

Our study finds that NaCl treatments had a significant influence on the proportion of root length for 0.1–0.2 mm and 0.5–2.0 mm diameter roots (Table 5) but had no significant effect on the proportion of root length for 0–0.1 mm and>2.0 mm diameter roots. Previous studies observed an increase in the percentage of thick roots and a decreased percentage of fine roots under saline conditions in black spruce and jack pine [45]. Our findings were not consistent with those of previous studies. The different effects of salinity on the proportion of roots of different diameters can also depend on plant species or the tested soil type.

**Table 5:** Proportion of root length by diameter to experimental factors, Aeration volume (A) and Salinity level (S)

treatments	Proportion (%) of root length by diameter (mm)					
	0–0.1	0.1–0.2	0.2–0.5	0.5–1.0	1.0–2.0	>2.0
CK	47.85ab	22.51bcd	21.56ab	6.39abc	1.47abcd	0.21b
S_1_A_0_	48.65ab	22.20bcd	22.00ab	5.72bc	1.21bcd	0.23ab
S_1_A_1_	44.12b	20.99 cd	23.18a	8.57a	2.57ab	0.56ab
S_1_A_2_	49.40ab	21.07 cd	20.32ab	6.90abc	1.93abc	0.38ab
S_1_A_3_	50.55ab	21.07 cd	19.81b	6.48abc	1.76abcd	0.33ab
S_2_A_0_	45.72ab	25.59a	21.84ab	5.23c	1.29bcd	0.32ab
S_2_A_1_	45.91ab	20.18d	22.33ab	8.04ab	2.81a	0.73a
S_2_A_2_	47.77ab	22.19bcd	20.83ab	6.84abc	2.04abc	0.32ab
S_2_A_3_	51.04ab	21.38 cd	19.39b	6.00abc	1.75abcd	0.45ab
S_3_A_0_	–	–	–	–	–	–
S_3_A_1_	58.24	19.24	17.30	3.93	0.86	0.43
S_3_A_2_	48.66ab	24.29ab	21.63ab	4.43 cd	0.69 cd	0.29ab
S_3_A_3_	57.80a	23.19bc	15.92c	2.23d	0.48d	0.38ab
F-value						
Aeration volume (A)	6.210**	9.107**	8.528**	5.599**	4.448*	2.502
Salinity level (S)	1.470	6.145**	0.844	6.333**	3.492*	0.257
A × S	1.531	2.604	2.029	0.292	0.026	0.190

Note: Values followed by different small letters in the same column meant significant differences at p < 5%. ANOVA F-value for main and interaction effects were not significant (ns) or significant at ≤5% (*) and ≤ 1% level (**)

Many studies showed a strong negative correlation between salt concentration and photosynthesis [27, 46, 47]. Bethke’s research [23] found that both Na^+^ and Cl^−^ have a direct effect on the photosynthetic apparatus because they reduce the efficiency of ribulose-1 5-bisphosphate carboxylase (Rubisco) in the Calvin cycle. Similarly, our results showed that salt can restrain the growth of tomato (Table 1). Photosynthesis was reduced with increasing concentrations of NaCl (Fig. 4). This conclusion is consistent with previous studies in tomato [48] and tobacco [9].

High *G*_s_ in plants means higher CO_2_ diffusion into the leaf, thereby favoring higher photosynthetic rates, resulting in higher fruit yields [49]. Under salinity stress, a distinct decrease in the *G*_s_ and consistent reductions in *P*_n_ and *T*_r_ lead the plant to obtain the maximum water use efficiency [50]. The photosynthesis of tomato was deeply reduced, so the stressed plants had a lower amount of fixed carbon (CO_2_) to utilize for plant growth. Our results suggest that NaCl stress decreases *G*_s_ and photosynthetic activity, which may inhibit whole-plant growth. Koyro [50] suggested that this behavior is an adaptive mechanism to cope with salt, especially during periods of high transpiration. The moderate salt level (74 mM NaCl) reduced the total chlorophyll by 27.3% compared with the control plants at 108 DAT (Table 4). It also depressed the *P*_n_ by 46.6, 64.2 and 63.2% compared with the control plants at 46, 108 and 150 DAT, respectively (Fig. 4). These results indicate that NaCl stress depressed the photosynthetic capacity of leaves, which might lead to the suppression of photosynthate from leaves to other tissues. This tendency is more pronounced at higher salt levels. We also found that tomato could not survive at 120 mM NaCl. Concentrated salt depositions in the soil generate a low water potential zone in the soil, making it increasingly difficult for the plant to acquire both water and nutrients [1]. Additionally, the accumulated toxins often lead to cell death, which might also contribute to the death of the whole plant [51].

### Soil aeration strongly promotes root growth, *P*_n_ and chlorophyll content of tomato plants under NaCl salinity conditions

Soil aeration is an important but often unheeded site factor for plant growth. Like salinity, hypoxia often reduces transpiration and nutrient uptake and inhibits plant growth [52]. Previous research has shown that tomato roots are especially vulnerable to low levels of oxygen in soil [53, 54]. Soil aeration can effectively promote redoxase enzyme activity and root metabolism, thereby enhancing nutrient absorption and accelerating the growth and yield of plants [31, 55, 56]. To date, there are no reports in the literature specifically examining the sensitivity of tomato plants in salinized soil to aeration and how this may impact root growth and photosynthetic characteristics. However, such information would have high practical value and would be especially important in greenhouses. As we know, soil aeration can speed up the gas exchange process and increase the oxygen content in soil. The high oxygen content in soil could ensure that the physiological activity of the root occurs smoothly [34, 57]. The basic physiology of high NaCl stress and soil compaction stress overlap with each other. A high NaCl concentration in the soil can alter the basic texture of the soil, resulting in decreased soil porosity and consequently reduced soil aeration and water conductance. Plants utilize several mechanisms to reduce the negative effects of hypoxia and salt stress. These include signaling pathways controlled by ethylene, abscisic acid (ABA) and reactive oxygen species [58]. For plants to survive, one of the fastest responses to environmental stress is the closing of stomatal pores on the leaf surfaces. Stomatal closure due to ABA accumulation in leaf tissues has been a prolific research field for many years. Stomata regulate the uptake of CO_2_ for photosynthesis and the loss of water vapor during transpiration [59]. Stella demonstrated that leaf and stem dry matter reduction can be associated with a reallocation of photosynthates to the roots mediated by ABA signaling [60]. ABA induces the production of hydrogen peroxide (H_2_O_2_) in guard cells, which in turn activates Ca^2+^ channels to pump Ca^2+^ across the plasma membrane, increasing cytosolic Ca^2+^ and resulting in Ca^2+^-dependent stomatal closure. The rapid catabolism of ABA in leaves prevents the accumulation of ABA, allowing stomata to open as soon as water conditions return to favorable levels [61, 62]. However, there are no reports in the literature specifically examining the sensitivity of tomato plants in salinized soil to aeration. Previous studies have shown that “reduced photosynthesis and stomatal closure are common responses to flooding-induced deficiencies in soil oxygen” [63]. Else’s research [64] also found that “stomatal closure has been linked to a rise in the concentration of bulk aba in the leaves under flooding”. Our results showed that NaCl stress significantly decreased root length, surface area, volume, *P*_n_ and *G*_s_. These variables were enhanced by artificial soil aeration. Therefore, we speculated that soil aeration may decrease the concentration of ABA. Lower concentrations of ABA can promote *G*_s_ and *P*_n_, which may promote whole plant growth.

In this experiment, the soil texture was clay loam. Plastic film mulch and no drainage holes at the bottom of the plastic barrel reduced the aeration of the soil. Rhizosphere hypoxia negatively influenced shoot and root growth, which is consistent with previous research showing that hypoxia decreases the growth of roots and shoots [21]. Root respiration has been shown to be improved by aeration when plants are grown in clay loam. Root morphology (total root length, surface area, volume and number of forks) was significantly enhanced by aeration at each salinity level. This positive effect of aeration on root morphology was mainly evident in the S_1_ treatment. This was mainly because both hypoxia and salinity conditions cause increased Na^+^ and Cl^−^ concentrations in the shoot, due initially to increased rates of transport. These increased concentrations in the shoots have adverse effects on plant growth and survival [21]. The positive effect of aeration on root morphology at each salinity level in the clay loam soil may be due to the alleviation of O_2_ deficiency in the rhizosphere.

It was found that root characteristics had a significant positive correlation with *P*_n_, *G*_s_ and Chl. a (Table 6). In this study, both hypoxia and salinity depressed plant growth. The two factors easily lead to a reduction in the root system and photosynthetic characteristics. Our previous studies have shown that soil aeration increased the root length and surface area of muskmelon in clay soils [34]. Salt stress increases abscisic acid synthesis in the root zone, but a favorable soil gas environment reduces it while reducing the abscisic acid contents in leaves [65]. There was a negative correlation between abscisic acid and stomatal resistance. In this study, it is reasonable to infer that root zone aeration might lead to the reduction of abscisic acid synthesis in roots and transport from roots to leaves, and the reduction of abscisic acid in leaves can directly promote *G*_s_ (Table 6), thereby providing enough raw material (CO_2_) for the photosynthesis of tomato plants. In addition, Chl. a, root length and surface area increasing with increased aeration might improve the *P*_n_ and uptake of water and nutrients at each salinity level.

**Table 6:** Simple correlation analysis between root characteristics, photosynthetic characteristics and chlorophyll content of potted tomato

Factor	Root length	Root surface area	Root volume	forks	*P*_n_	*G*_s_	*C*_i_	*T*_r_	*IWUE*	Chl. a	Chl. b	Chl. (a + b)
Root length	1											
Root surface area	0.991^**^	1										
Root volume	0.961^**^	0.989^**^	1									
forks	0.988^**^	0.992^**^	0.975^**^	1								
*P*_n_	0.707^*^	0.696^*^	0.670^*^	0.657^*^	1							
*G*_s_	0.758^**^	0.742^**^	0.710^**^	0.719^**^	0.962^**^	1						
C_*i*_	−0.517	−0.500	−0.474	−0.457	−0.809^**^	−0.740^**^	1					
*T*_r_	0.388	0.444	0.496	0.383	0.622^*^	0.485	−0.277	1				
*IWUE*	0.657^*^	0.605^*^	0.536	0.616^*^	0.709^**^	0.806^**^	−0.770^**^	−0.083	1			
Chl. a	0.764^**^	0.773^**^	0.769^**^	0.747^**^	0.882^**^	0.822^**^	−0.830^**^	0.616^*^	0.635^*^	1		
Chl. b	0.445	0.484	0.527	0.447	0.475	0.397	−0.760^**^	0.285	0.434	0.743^**^	1	
Chl. (a + b)	0.713^**^	0.731^**^	0.741^**^	0.700^*^	0.811^**^	0.742^**^	−0.856^**^	0.552	0.611^*^	0.980^**^	0.862^**^	1

Note: ^*^ represents a significant difference (*p* < 0.05), and ^**^ represents an extremely significant difference (*p* < 0.01)

## Conclusions

Our results demonstrated the following: (1) Aeration volume and salinity affected the total length, surface area, volume and number of forks of potted tomato plant root systems. The 4 root parameters increased with the increase in aeration volume under each NaCl stress level, and all these parameters decreased with the increase in NaCl stress; the effect was stronger for the fine-diameter roots. (2) NaCl stress significantly decreased *P*_n_, *G*_s_ and chlorophyll a and b contents. Aeration volume tended to increase these parameters of the potted tomato under NaCl stress conditions. Nevertheless, aeration volume did not markedly influence the contents of chlorophyll a and b. (3) Aeration effectively reduced the death rate of potted tomato plants under high salinity stress conditions (120 mM NaCl). The results proved that soil aeration can promote root growth and increase the photosynthetic rate, thus promoting plant growth and reducing the death rate under NaCl stress conditions.

## Methods

### Site description, plant and soil details

This study was carried out in a greenhouse of a vegetable farm (34°16′N, 108°04′E) at Northwest A&F University, Yangling, Shaanxi Province, West China, from October 2014 to March 2015. The climate of Yangling is semiarid with an average of 210 frost-free days. The rainfall is between 550 and 650 mm, and the annual sunshine time was 2164 h. The original source of the tomato was New Horizon Facilities Agricultural Development Co. Ltd., Northwest A&F University, China. The tomato cultivar “Fen-Yu-Yang-Gang” was sown in commercial seedling plugs. The tested soil was sourced from the surface (0–20 cm depth) of an experimental field at Northwest A&F University (34°16′N, 108°04′E). All the processes are legitimate. The soil texture was clay loam (sand 24.5%; silt 33.2%; and clay 42.3%; classified as Inceptisol based on the U.S. soil taxonomy). The soil properties were as follows: electrical conductivity 364.2 μS/cm, redox potential − 53.4 mV, bulk density 1.35 g cm^− 3^, field capacity 26.6% (by weight), pH 7.91. The ion concentrations of the tested soil were: 3.5 mM K^+^, 6.3 mM Na^+^, 11.1 mM Ca^2+^, 4.7 mM Mg^2+^, 6.4 mM SO_4_^2−^, 27.2 mM NO_3_^−^, and 4.6 mM Cl^−^.

### Experimental design

Tomato seeds were sown in commercial seedling plugs and transplanted to 104 experimental pots with seedlings of the same growth vigor after 20 days. Fertilizers were applied as basal fertilizer and evenly mixed into the soil at the commencement of the experiment. Before transplanting, carbendazim was used as an antiseptic, and 28 g/pot of decomposed organic manure (pig and sheep manure), 13.6 g/pot of diammonium phosphate (N 18%, P_2_O_5_ 46%) and 6 g/pot of compound fertilizer (N 18%, P_2_O_5_ 15%, K_2_O 12%) were mixed uniformly as basal fertilizer in the soil. Mulching film and 3.5 l of water were added to each pot on the transplanting day. After transplanting, 0.8 l of water was added to each pot every 4 days. Plastic barrels had an upper inner diameter of 28.5 cm and a bottom inner diameter of 21 cm. We placed a plastic hose with an inner diameter of 6 mm in spiral form at the bottom of the plastic barrel. The hose has eight special air outlets in its wall; each hole’s diameter is 2 mm and every three holes’ interval is 10 cm. The pipe was connected to an air compressor that provided aeration once every 2 days. To prevent clogging, the air outlets and soil were separated by gauze and then the soil was loaded layer by layer until there was 13 kg soil in the barrel.

**Fig. 5:**
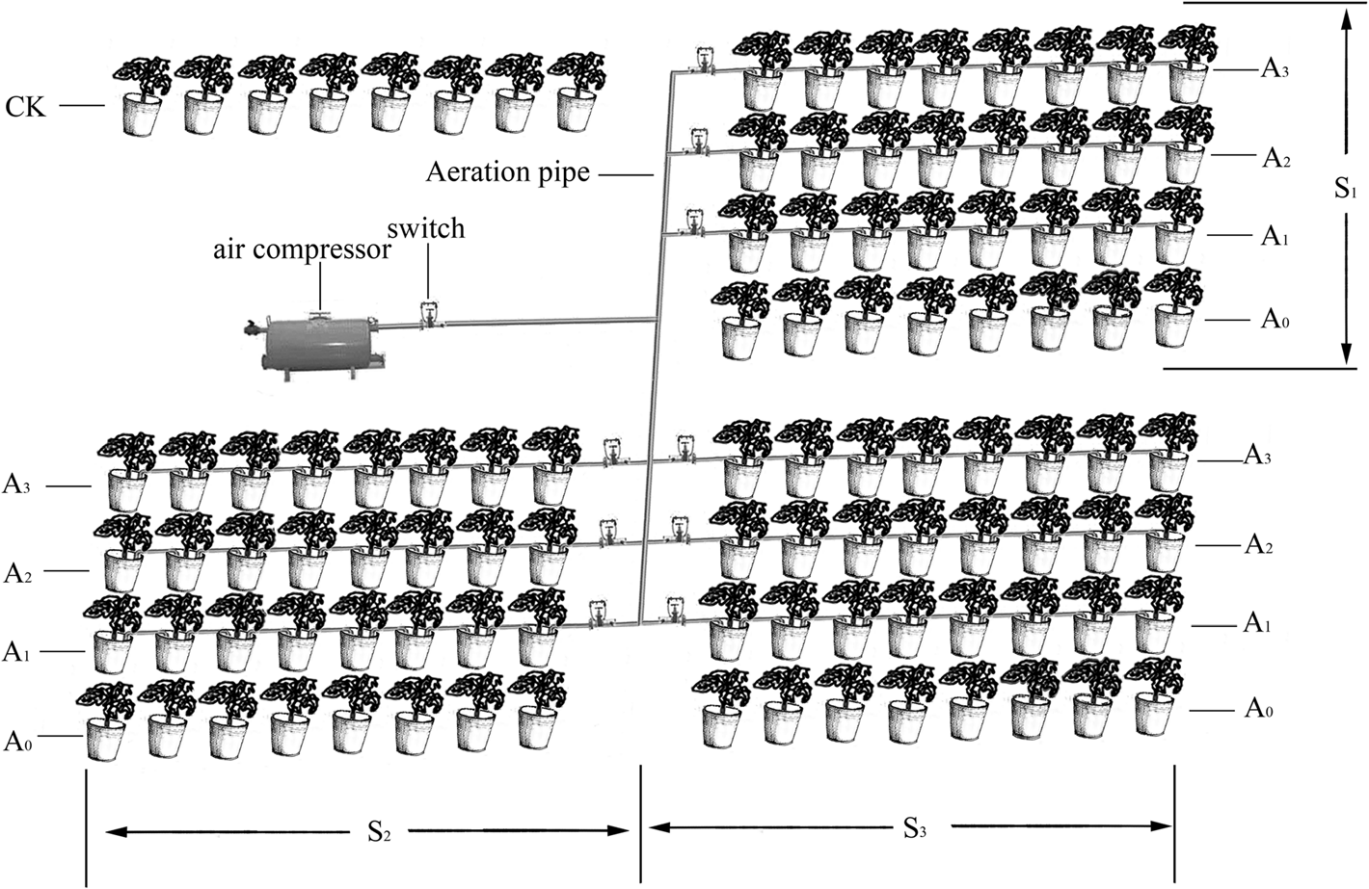
Sketch map of potted tomato plants at different NaCl stress and soil aeration levels. Notes: A0, A1, A2, and A3 represent aeration volume are non-aeration, 2.3, 4.6 and 7.0 L/pot, respectively. S1, S2 and S3 represent light, moderate and heavy NaCl stress, respectively. No NaCl stress with non-aeration served as control and was identified by the designation CK

The experimental design was treated as a 3 (salinity levels) × 4 (aeration volumes) full factorial design versus nonsaline soil with 8 replicates for statistical analyses (Fig. 5). For NaCl stress treatment, plants were treated with S_1_ (light NaCl stress), 29 mM Na^+^, 28 mM Cl^−^; S_2_ (moderate NaCl stress), 75 mM Na^+^, 74 mM Cl^−^; S_3_ (heavy NaCl stress), 121 mM Na^+^, 120 mM Cl^−^. Every 2 days, air was injected into the pots through the pipe by air compressor at approximately 4 to 6 PM during the whole growing season.

The air discharge rate of each pot was approximately 0.20 L/min. The aeration volume was controlled by aeration time. Four aeration volumes (A) were set for each NaCl-salt level: A_0_, A_1_, A_2_, and A_3_ represent non-aeration, 0.5 (2.3 L/pot), 1.0 (4.6 L/pot) and 1.5 (7.0 L/pot) times the standard aeration volume (4.64 L/pot), respectively. Non-NaCl stress (6.3 mM Na^+^, 4.6 mM Cl^−^) with no aeration served as a control and was identified by the designation CK. Each treatment was replicated 8 times. Using 50% of soil porosity as the standard aeration volume (4.64 L/pot) [57, 66], the porosity (n) of the silty clay loam soil in the pots was estimated as n = (1-ρ_b_/ρ_p_) × 100%; ρ_b_: dry bulk density, ρ_p_: particle density. Using the measured ρ_b_ = 1.35 g cm^− 3^ and taking ρ_p_ = 2.65 g cm^− 3^ gives an estimated *n* = 49.1%. All agronomic management measures taken during the growth period of tomato such as fertilization, agricultural chemical spraying, etc. were consistent with local production practice.

### Test items and methods

Growth rate (GR) was calculated as $\mathrm{GR}=\frac{{\mathrm{M}}_2-{\mathrm{M}}_1}{{\mathrm{T}}_2-{\mathrm{T}}_1}$, from two measurement times T_2_ and T_1_, and plant height or stem diameter was M_2_ and M_1_. The plant height and stem diameter in each plot were measured at 0, 30, 50, 70, and 90 days after transplantation (DAT) using a steel ruler and electronic digital calipers. All measurements were performed before topping.

Net photosynthetic rate (*P*_n_), stomatal conductance (*G*_s_), transpiration rate (*T*_r_) and intercellular CO_2_ concentration (*C*_i_) were measured on healthy attached leaves between 9:00 and 11:00 AM on bright, sunny days during the flowering and fruit setting stage (46 DAT), fruit enlargement stage (108 DAT) and fruit ripening stage (150 DAT). The leaf position was the same for all plants, and leaves were adequately lighted. These measurements were made with a LI-6400 portable photosynthesis measurement system (Li-Cor, Inc., Lincoln, Nebraska, USA) with an internal red/blue LED light source (Li-6400-02B, Li-Cor, Lincoln, Nebraska, USA) with irradiance set at 600 μmol·m^− 2^ s^− 1^. The open flow gas exchange flow rate was 500 μmol·s^− 1^. The instantaneous water use efficiency (*IWUE*) was calculated according to *IWUE* = *P*_n_/*T*_r_ [67, 68]. Chlorophyll a and b were measured during the fruit setting stage (46 DAT) and fruit enlargement stage (108 DAT). A 95% ethanol solution was used to extract the pigments. Chlorophyll a and b in the extract were measured by absorbance spectrophotometry (at 665, 649, and 470 nm, respectively) and used to calculate the content of chlorophyll a and b and total chlorophyll (a + b) content in leaves [69].

After the maturation period (155 DAT), the aboveground parts of the tomato plants were removed, and the soil in the pot was placed on a 100-mesh steel sieve, soaked, and gently washed to separate the roots from the soil. The roots were then dried with absorbent paper. The roots were placed on a transparent tray filled with water to a depth of 10 mm. The trays were scanned with an Epson Perfection V700 scanner to obtain a grayscale TIFF image. This image was then analyzed with the WinRHIZO Pro image processing system (Regent Instruments Inc., 2672 Chemin Sainte-Foy, Quebec City, Quebec G1V 1 V4, Canada) to obtain total root length, surface area, volume, and their distribution over diameter ranges (D in mm) of 0<D ≤ 0.1, 0.1<D ≤ 0.2, 0.2<D ≤ 0.5, 0.5<D ≤ 1.0, 1.0<D ≤ 2.0, and 2<D.

### Data analysis

SPSS 22.0 software (IBM Crop., Armonk, New York, NY, USA) was used to analyze the experimental data. Multiple comparisons using Duncan’s new multiple-range test were completed whenever the ANOVA indicated significant differences (*P* ≤ 5%). Root morphology, plant height, stem diameter and chlorophyll content were analyzed by two-way ANOVA with the factors aeration volume treatment, NaCl stress and the interactions of aeration volume treatment × NaCl stress. Figures were constructed using the graphing software Origin-Pro 8.5 (Origin Lab Corporation, Northampton, MA, USA) and Photoshop CS 5 (Adobe Systems Inc., San Jose, California, USA).

## Data Availability

The datasets used and/or analyzed during the current study are available from the corresponding author on reasonable request.

## Abbreviations

ABA: Abscisic acid
Chl. a: Chlorophyll a
Chl. b: Chlorophyll b
Chl. t: Total chlorophyll
*C*_i_: Intercellular CO_2_ concentration
*G*_s_: Stomatal conductance
*IWUE*: Instantaneous water use efficiency
mM: mmol/L
*P*_n_: Net photosynthetic rate
*T*_r_: Transpiration rate
